# Supplementary material for: FOXO1 Confers Maintenance of the Dark Zone Proliferation and Survival Program and Can Be Pharmacologically Targeted in Burkitt Lymphoma
Source: Cancers (Basel). 2019 Sep 25;11(10):1427. doi: 10.3390/cancers11101427 (PMC6826697; doi:10.3390/cancers11101427)
Supplement: Supplementary file 1 [file cancers-11-01427-s001.zip › cancers-581251-supplementary/Supplementary Materials-final/cancers-581251-supply methods and Table S1 and Figure S1-S6.docx]

1. Supplemental Methods

1.1. Cell lines and treatment

BL cell lines (Ramos, BL-41, Namalwa, Daudi, Jiyoye, Raji) were cultured in RPMI1640 medium (Life Technologies) supplemented with 15 % fetal calf serum (PAN-Biotech, Aidenbach, Germany), 2 mM L-glutamine, 100 U/mL penicillin, 100 µg/mL streptomycin, and 50 µM monothioglycerol at 37°C and 5 % CO_2_. The cHL cell line L-428 was cultured in a similar way, with the only difference that the medium was supplemented with 10 % fetal calf serum. The cHL cell line U-HO1[^1^](#_ENREF_1) was cultured in RPMI1640/Iscove medium (1:4) supplemented with 20 % FBS, glutamine and antibiotics.

Cell lines were authenticated by short tandem repeat (STR) DNA typing using GenomeLab^TM^ GeXP Genetic Analysis System (Sciex, Darmstadt, Germany) and GenomeLabHuman STR primer set (Beckman Coulter, Brea, CA, USA) or experiments were performed right after receipt from DSMZ. STR profiles were analyzed using the “Online STR Analysis” tool provided by DSMZ (comprehensive DSMZ database of STR cell line profiles, [www.dsmz.de](http://www.dsmz.de)) and the ExPASy bioinformatics resource portal database “Cellosaurus” (<https://web.expasy.org/cellosaurus/>; RRID: SCR_013869).

Mycoplasma contamination was controlled using the Mycoplasma Detection Kit-Quick-Test (Biotool.com, Absource Diagnostics, Munich, Germany).

1.2. Vectors

1.2.1. shRNA-mediated knockdown

The shRNA for *FOXO1* was selected from the DECIPHER shRNA Library, Human Module I (sh_hFOXO1.H-10381, Cellecta, https://www.cellecta.com/) obtained from BioCat (Heidelberg, Germany). The shRNA target sequences for *FOXO1/3/6* and *MYB* were selected from Broad Institute RNAi consortium shRNA library (https://www.broadinstitute.org/rnai-consortium/rnai-consortium-shrna-library). As a negative control we used a scramble sequence that does not have targets in the human genome.[^2^](#_ENREF_2) Corresponding oligonucleotides were assembled and cloned into the BsbI and EcoRI cutting sites of pRSI12-U6-sh-UbiC-TagRFP-2A-Puro (Cellecta, <https://www.cellecta.com/>) with help of the Gibson Assembly method (NEB, Ipswich, MA, USA). ShRNA target sequence as well as amplification primers are listed in the table below.

| **Vector** | **Broad Institute Clone ID** | **Target sequence** | **Amplification primers** |
| --- | --- | --- | --- |
| pRSI12-FOXOsh1/3/6 | TRCN0000020707 | CGTGCCCTACTTCAAGGATAA | for-5’-ACCGGCGTGCCCTACTTCAAGGATAAG TTAATATTCATAGCTTATCCTTGAAGTAGGGCACGTTTTTTG  rev-5’-AATTCAAAAAACGTGCCCTACTTCAAG GATAAGCTATGAATATTAACTTATCCTTGAAGTAGGGCACGC |
| pRSI12-MYBsh#1 | TRCN0000288659 | GCTCCTAATGTCAACCGAGAA | for-5´-ACCGGGCTCCTAATGTCAACCGAGAAG TTAATATTCATAGCTTCTCGGTTGACATTAGGAGCTTTTTTG  rev-5´-AATTCAAAAAAGCTCCTAATGTCAACC GAGAAGCTATGAATATTAACTTCTCGGTTGACATTAGGAGCC |
| pRSI12-MYBsh#2 | TRCN0000295917 | ACTATTCCTATTACCACATTT | for-5´-ACCGGACTATTCCTATTACCACATTTG TTAATATTCATAGCAAATGTGGTAATAGGAATAGTTTTTTTG  rev-5´-AATTCAAAAAAACTATTCCTATTACCA CATTTGCTATGAATATTAACAAATGTGGTAATAGGAATAGTC |

1.3. Oxidative Stress Measurement

Vectors for oxidative stress measurement were generated by cloning Grx1-roGFP2 from pLPCX mito into XbaI and BamHI cutting sites of pRSI12-scr or pRSI12-F1sh vector. By cloning the RFP ORF from pRSI12 has been removed.

| **Vector** | **Template vector** | **Amplification primers** |
| --- | --- | --- |
| pRSI12-Grx1-roGFP2-scr | pLPCX mito Grx1-roGFP2 (gift from Tobias Dick, Addgene plasmid #64977) | for-5’-CGCTGTGATCGTCACTTGGTATGGCCTCCAC  TCGTGTC  rev-5’-GAAGGCTTCCTCTGCCCTCGGACTTGTACA  GCTCGTCCATG |
| pRSI12-Grx1-roGFP2-FOXO1sh |  |  |

1.4. Expression Vectors

For gene expression we used the SF-LV vector[^3^](#_ENREF_3) (provided by K.L. Rudolph, Leibniz Institute for Age Research, Germany). The ORFs of genes of interest were cloned into the NotI cutting site of SF-LV-cDNA-EGFP by the Gibson Assembly method (NEB). Template vectors as well as amplification primers are listed in the table below.

| **Vector** | **Template vector** | **Amplification primers** |
| --- | --- | --- |
| SF-LV-MYB | pINDUCER21-MYB (gift from George Daley, Addgene plasmid #51305) | for-5´-TAATTAACTCGAGTTAACGCACCATGGCC CGAAGACCC  rev-5´-TTAGGGGGGGGGGAGGGAGAGGGGGCCTA CATGACCAGCGTCCGG. |

SF-LV-FOXO1(A3)ER expressing a constitutively active version of FOXO1 was constructed by cloning FOXO1(A3) from pBabe-FOXO1(A3)ER (obtained from T.G. Unterman, Chicago, Illinois) into the NotI cutting site of SF-LV-cDNA-EGFP. SF-LV-FOXO1(WT) was constructed by cloning FOXO1(WT) from pHIV-dTomato-FOXO1(WT) into NotI cutting site of SF-LV-cDNA-EGFP.

1.5. Rescue vectors

The plasmids for performing rescue experiments from *FOXO1* knockdown were constructed by cloning the respective insert into the PshAI and SalI cutting sites of pRSI12-FOXO1shRNA. Rescue construct for FOXO1 was constructed by cloning wtFOXO1-wobbled into pRSI12-FOXO1shRNA. Vector expressing wtFOXO1-wobbled was designed as described.[^4^](#_ENREF_4)

| **Vector** | **Template vector** | **Amplification primers** |
| --- | --- | --- |
| pRSI12-F1sh-F1wob | pBabe-FOXO1ER-wobbled | for-5´-CCCTTCCGGGATGGCCGAGGCGCCTCAG  rev-5’-GTAATCCAGAGGTTGATTGTCAGCCTGACACCCAGCTATGT GTCGTTGTCTTGAC |
|  | pRSI12-F1sh | for-5´-GGAGGCCGACAAAGAGACCTACGTCGAGCAGCACGAGG  rev-5’-CCTCGGCCATCCCGGAAGGGCCGGGATT |
| pRSI12-F1sh-CCND3 | SF-LV-CCND3 | for-5’-CCTTCCGGGATGGAGCTGCTGTGTTGC  rev-5’-TGTAATCCAGAGGTTGATTGCTACAGGTGTATGGCTGTG |
|  | pRSI12-F1sh | for-5’-GGAGGCCGACAAAGAGACCTACGTCGAGCAGCACGAGG  rev-5’-GCAGCTCCATCCCGGAAGGGCCGGGATT |

1.6. CRISPR/Cas9 vectors

The single guide RNA sequences targeting the *FOXO1* gene (sgF1.1, sgF1.2) were selected from Broad Institute GPP Web Portal using the sgRNA design tool (<https://portals.broadinstitute.org/gpp/public/analysis-tools/sgrna-design>). Subsequently, corresponding oligonucleotides were assembled and cloned into the BsmBI cutting sites of pL-CRISPR.EFS.tRFP (gift from Benjamin Ebert, Addgene plasmid #57819). As negative control a non-targeting sgRNA (NT) without targets in the human genome was used (Doench et al. Nat Biotechnol. 2016 Feb;34(2):184-191.) The sgRNA target sequences are listed in the table below.

| **Vector** | **Sequence** |
| --- | --- |
| pL-CRISPR-NT | GTATTACTGATATTGGTGGG |
| pL-CRISPR-sgFOXO1.1 | GAGTTGGACTGGCTAAACTC |
| pL-CRISPR-sgFOXO1.2 | GGAGTTTAGCCAGTCCAACT |

Two sgRNAs targeting the *miR-150* gene were designed using the sgRNA Designer (CRISPRko, Broad Institute GPP Web Portal (https://portals.broadinstitute.org/gpp/public/analysis-tools/sgrna-design) and CRISPR RGEN Tools Cas-OFFinder (http://www.rgenome.net/cas-offinder/) webtools such that efficient cutting by both sgRNAs would result in deletion of the majority of the pri-miR-150 sequence. To create the lentiCRISPR_miR-150_CR2CR5 vector, a minigene containing the sequences of both sgRNAs as well as the gRNA scaffold sequence for the first sgRNA and the H1 promoter sequence upstream of the second sgRNA was designed. Upon processing of the minigene with the restriction enzyme BbsI, it was cloned into the BsmBI cutting site of the lentiCRISPR-EV-GFP vector (kind gift from Ryan M. O’Connell). As negative control, the lentiCRISPR_NTCR1CR2 vector was created containing two non-targeting sgRNAs without targets in the human genome, which were taken from the Human GeCKO v2 library (Addgene Pooled Library #1000000048 and #1000000049). The sgRNA as well as the minigene sequences are listed in the table below.

| **Vector** |  | **Sequence** |
| --- | --- | --- |
| lentiCRISPR_miR-150_CR2CR5 | sgRNA CR2 | CCCAGCACTGGTACAAGGGT |
|  | sgRNA CR5 | CCGGCACCGGCAGGCCCCAA |
|  | minigene | GAAGACaaCACCGCCCAGCACTGGTACAAGGGTGTTTTAGAGCTAGAAATAGCAAGTTAAAATAAGGCTAGTCCGTTATCAACTTGAAAAAGTGGCACCGAGTCGGTGCTTTTTTCCCAACGATGTCAAGAATTGGAACGCTGACGTCATCAACCCGCTCCAAGGAATCGCGGGCCCAGTGTCACTAGGCGGGAACACCCAGCGCGCGTGCGCCCTGGCAGGAAGATGGCTGTGAGGGACAGGGGAGTGGCGCCCTGCAATATTTGCATGTCGCTATGTGTTCTGGGAAATCACCATAAACGTGAAATGTCTTTGGATTTGGGAATCTTATAAGTTCTGTATGAGACCACTTGCACCGCCGGCACCGGCAGGCCCCAAGTTTCAGTCTTC |
| lentiCRISPR_NTCR1CR2 | sgRNA CR1 | ACGGAGGCTAAGCGTCGCAA |
|  | sgRNA CR2 | ATCGTTTCCGCTTAACGGCG |
|  | minigene | GAAGACaaCACCGACGGAGGCTAAGCGTCGCAAGTTTTAGAGCTAGAAATAGCAAGTTAAAATAAGGCTAGTCCGTTATCAACTTGAAAAAGTGGCACCGAGTCGGTGCTTTTTTCCCAACGATGTCAAGAATTGGAACGCTGACGTCATCAACCCGCTCCAAGGAATCGCGGGCCCAGTGTCACTAGGCGGGAACACCCAGCGCGCGTGCGCCCTGGCAGGAAGATGGCTGTGAGGGACAGGGGAGTGGCGCCCTGCAATATTTGCATGTCGCTATGTGTTCTGGGAAATCACCATAAACGTGAAATGTCTTTGGATTTGGGAATCTTATAAGTTCTGTATGAGACCACTTGCACCGATCGTTTCCGCTTAACGGCGGTTTCAGTCTTC |

1.7. CRISPR mutational analysis

For evaluating the mutation efficiency of the *FOXO1* knockout constructs, RFP^+^ cells expressing pL-CRISPR-NT, pL-CRISPR-sgFOXO1.1 or pL-CRISPR-sgFOXO1.2 were sorted 7 days post transduction and DNA was isolated using the DNeasy Blood and Tissue Kit (QIAGEN, Hilden, Germany). Target site of both sgRNAs within the *FOXO1* gene was amplified with the following primers: for-5´-GCTGCGTTCTCCCCCTCTTG and rev-5´-CGCTCTCCTCCAGCAAGCTC and sequenced by Eurofins Genomics (Ebersberg, Germany).

Sequence analysis by TA cloning was performed by cloning the amplified target site of both sgRNAs into pGEM-T Easy vector (Promega, Madison, WI, USA) and sequenced by Eurofins Genomics using the T7 primer for-5’-TAATACGACTCACTATAGGG.

For confirming the editing of the *miR-150* gene, GFP^+^ cells expressing lentiCRISPR_miR150_CR2CR5 or lentiCRISPR_NTCR1CR2 were sorted 4 days post transduction and DNA was isolated using the DNeasy Blood and Tissue Kit (QIAGEN, Hilden, Germany). The region including the *miR-150* gene was amplified with the following primers: for-5´-CATCACACAGAGGTGGGGAC and rev-5´- TGCAGTTTCTGCGACTCAGG using an annealing temperature of 59°C. The PCR product was run on a 2 % agarose gel. The non-edited gene yields a PCR product of 443 bp, whereas removal of the majority of the *miR-150* sequence results in a 366 bp PCR product.

1.8. FOXO1 and CCND3 mutational analysis

The mutational status of the first exon of the *FOXO1* gene was analyzed by isolating genomic DNA from BL cell lines using DNeasy Blood and Tissue Kit (QIAGEN), followed by amplification with help of Phusion High-Fidelity DNA polymerase (NEB). Exon 1 was amplified with primers for-5’-ATCGCAGCGAAAGAAAACAT and rev-5’-CTTCTTGGCGCACTTTCTTT. The PCR products were purified by agarose gel electrophoresis followed by extraction of the amplified product with help of QIAquick Gel Extraction Kit (QIAGEN). The purified amplification products were sequenced by GATC Biotech (Konstanz, Germany) with the sequencing primer for-5´-TGTGTGAAAAACACCC.

The mutational status of the *CCND3* CDS was analyzed by isolation of RNA and cDNA generation as described previously,[^5^](#_ENREF_5) followed by amplification with help of Expand Long Template PCR System (Roche Applied Science, Penzberg, Germany). cDNA was amplified with primers for-5’- GCTGCCCGAGTATGGAGCTG and rev-5’- GTGGTGTGGTTCCTGGAGGC. The PCR products were purified with the QIAquick PCR Purification Kit (QIAGEN) and sequenced by GATC Biotec, with help of the same primers as used for amplification. *FOXO1* and *CCND3* sequences were analyzed with help of Lasergene software (DNASTAR, RRID: SCR_011854).

1.9. Immunoblotting

Cell lines were lysed in Laemmli buffer containing 6 M urea and 5 % 2-mercaptoethanol or SDS sample buffer (62.5 mM Tris-HCl (pH 6.8), 2 % SDS, 10 % Glycerin, 50 mM DTT, 0.01% bromphenol blue) and boiled for 5 - 10 minutes at 100 °C. For detection of phosphorylated proteins, lysates were sonicated 3 times for 5 seconds before boiling. Lysates were separated by SDS-PAGE and electrophoretically transferred to a 0.45 µm nitrocellulose membrane, with the exception of detection of HIST3H3 for which a 0.2 µm membrane was used. The membrane was blocked for 15 minutes at 37 °C in 5 % non-fat dried milk/TBS and primary antibodies were incubated at 4 °C overnight. Next day, the membrane was washed twice in TBS-T (0.5% Tween-20) and once in TBS (5 minutes per washing step). Horseradish peroxidase-conjugated secondary antibody was diluted 1:5000 - 1:10000 (goat anti-rabbit, #31460, Thermo Fisher Scientific, Waltham, MA, USA; RRID: AB_228341) or 1:5000 (goat anti-mouse: 1:10 000, #sc-2005, Santa Cruz Biotechnology, Dallas, TX, USA; RRID: AB_631736) in 5 % non-fat dried milk/TBS-T or TBS-T only, and the membrane was incubated for 1 h at RT in these solutions. Washing of the membrane was repeated as before and protein bands were visualized by addition of SuperSignal West Dura Extended Duration Substrate (Thermo Fisher). TUBB, GAPDH, ACTB, HIST3H3 or RELA expression levels were used as loading controls.

| **Primary antibody** | **Species** | **Product number** | **Dilution factor** | **RRID** |
| --- | --- | --- | --- | --- |
| FOXO1 | rabbit | #2880, Cell Signaling | 1:250 | AB_2106495 |
| HIST3H3 | rabbit | #07-690, Millipore | 1:50000 | AB_417398 |
| pAKT-T308 | rabbit | #13038, Cell Signaling | 1:1000 | AB_2629447 |
| pAkt-S473 | rabbit | #9271, Cell Signaling | 1:1000 | AB_329825 |
| pRELA | rabbit | #3033, Cell Signaling | 1:1000 | AB_331284 |
| RELA | rabbit | #sc-372, Santa Cruz | 1:1000 | AB_632037 |
| MYB | rabbit | #12319, Cell Signaling | 1:1000 | AB_2716637 |
| CXCR4 | rabbit | #124824, Abcam | 1:500 | AB_10975635 |
| CCND3 | mouse | #2936, Cell Signaling | 1:1000 | AB_2070801 |
| Cl.CASP3 | rabbit | #9661, Cell Signaling | 1:1000 | AB_2341188 |
| CDKN1B | rabbit | #3686, Cell Signaling | 1:1000 | AB_2077850 |
| TOM20 | rabbit | #11415, Santa Cruz | 1:250 | AB_2207533 |
| TUBB | rabbit | #6046, Abcam | 1:100000 – 1:800000 | AB_2210370 |
| ACTB | rabbit | #sc-8432, Santa Cruz | 1:1000 | AB_626630 |

1.10. Quantitative reverse-transcription PCR (qRT-PCR)

Isolation of RNA, cDNA generation and RT-qPCR were performed as described previously.[^5^](#_ENREF_5) For ensuring target specificity, primers were analyzed with Primer-BLAST (<http://www.ncbi.nlm.nih.gov/tools/primer-blast/>; RRID: SCR_003095) and subsequently synthesized by biomers.net. Annealing temperature was set to 60°C and samples were measured in duplicates using the LightCycler 480 instrument (Roche). The relative expression of the target gene was calculated with help of the 2^-ΔΔCT^ method. RPL13A was used as housekeeping gene. All primer sequences are listed below.

| **RT-qPCR primers** | |
| --- | --- |
| RPL13A | for-5´-CGGACCGTGCGAGGTAT  rev-5´-CACCATCCGCTTTTTCTTGTC |
| FOXO1 | for-5´-TGGACATGCTCAGCAGACATC  rev-5´-TTGGGTCAGGCGGTTCA |
| AICDA | for-5´-GCATGGTCACCTTCAAGCTA  rev-5´-TTGCGTTTCCAGAAGATTTG |
| RAD51 | for-5´-GGTGAAGGAAAGGCCATGTA  rev-5´-CATCACTGCCAGAGAGACCA |
| RAD51AP1 | for-5´-GACTTCGGTGGACTCTGCTC  rev-5´-GGTGGGACCCATTTAGGTTT |
| IRF4 | for-5´-CAGTGGGCTGTTTCTGCTTA  rev-5´-CAAGTGGAGGTCTTTGGGAT |
| CCND3 | for-5´-GTGGCCACTAAGCAGAGGAG  rev-5´-CCCTTCAGGCTTAGATGTGG |
| BACH2 | for-5´-TGGACAACTTCTCCTGCCTT  rev-5´-GGTCTGAGGACAGGGCAATA |
| MYB | for-5´-ACCATTGCCGACCACACCAG  rev-5´-TGGCGAGGCGCTTTCTTCAG |
| TNSF10 | for-5´-TTCCTGAGCAACTTGCACTT  rev-5´-TGGACCATTTGTTTGTCGTT |
| U6 | for-5´- CTCGCTTCGGCAGCACA  rev-5´-AACGCTTCACGAATTTGCGT |

1.11. miRNA isolation, cDNA generation and RT-Q-PCR

miRNAs were isolated from 1 x 10^6^ cells using the PureLink™ miRNA Isolation Kit (Thermo Fisher). miRNA cDNA was generated with help of MystiCq® microRNA cDNA Synthesis Mix (Sigma-Aldrich) according to the manufacturer ́s instructions.

The expression of miR-150 was analyzedd using the MystiCq microRNA qPCR Assay Primer hsa-miR-150-5p (mature sequence: for-5´-UCUCCCAACCCUUGUACCAGUG) and MystiCq Universal Primer (Sigma-Aldrich). Annealing temperature was set to 60°C and samples were measured in duplicates using the LightCycler 480 instrument (Roche). The relative expression of the target gene was calculated with help of the 2^-ΔΔCT^ method. U6 was used as housekeeping gene.

1.12. Lentiviral transduction

HEK293T cells were transfected with the lentiviral plasmid of interest, the HIV-1 derived packaging plasmid p8.91 and the plasmid encoding the VSV-G envelope glycoproteins with help of polyethylenimine (PEI, Polysciences, Hirschberg an der Bergstraße, Germany). Viral supernatant was harvested 48 h and 72 h later and supplemented with 1 µg/ml polybrene. Cells were resuspended in viral supernatant and spinoculated at 2900 rpm for 2 h at 4 °C. Analysis was performed 3-5 days later.

1.13. Cell death analysis

For *FOXO1* knockdown experiments, RFP^+^ cells were sorted 4 days post transduction by the Core Facility “Fluorescent Activated Cell Sorting” (Medical Faculty of Ulm, Germany) using a FACSAria (BD Biosciences, San Jose, CA, USA) and recultured for 2 days at standard conditions. For FOXO1ER overexpression experiments, GFP^+^ cells were sorted using the S3e Cell Sorter (Bio-Rad, Hercules, CA, USA) and treated for 2 or 4 days with 4-OHT. 5 x 10^5^ - 1 x 10^6^ cells were washed with Annexin V binding buffer (0.1 M Hepes, pH 7.4, 1.4 M NaCl, 25 mM CaCl_2_), resuspendend in 76.5 µl staining solution (70 µl binding buffer, 5 µl Annexin V–FITC or Annexin V–APC (Immuno Tools, Friesoythe, Germany) and 1.5 µl PI (2 μg/mL, Sigma-Aldrich, St. Louis, MO, USA) and incubated light protected for 15 min at room temperature. After addition of 500 µl binding buffer, samples were analyzed using the FACSCalibur flow cytometer (BD Biosciences). Dead cells were defined as Annexin V or PI single or double positives. Specific Apoptosis (SA) was calculated as 100 * (Exp - Con) / (100 - Con). Dotplots were created using Flow Jo Software (RRID: SCR_008520).

1.14. Growth dynamics measurement by flow cytometry analysis

Flow cytometry for measurement of growth dynamics was performed using the FACSCanto (BD Biosciences). Percentages of GFP^+^, RFP^+^ or GFP^+^+RFP^+^ double positive cell populations were analyzed using the BD FACSDiva Software (RRID:SCR_001456) or Flow Jo Software (RRID: SCR_008520). For growth dynamics experiments, the percentage of transduced cells was measured every 3 days. First measurement was performed 4 - 5 days post transduction and the percentage of transduced cells was set as 100 %.

1.15. Cell cycle analysis

For *FOXO1* knockdown experiments, RFP^+^ cells were sorted 4 days post transduction by the Core Facility “Fluorescent Activated Cell Sorting” (Medical Faculty of Ulm, Germany) using a FACSAria (BD Biosciences). For FOXO1ER overexpression experiments, GFP^+^ cells were sorted using the S3e Cell Sorter (Bio-Rad) and treated for 2 or 4 days with 4-OHT. 5 x 10^5^ - 1 x 10^6^ cells were washed with PBS and fixed by adding 1 ml PBS and 3 ml ice-cold 70 % ethanol dropwise to the sample during vortexing. After incubation on ice for at least 1 h, cells were centrifuged, resuspended in PBS containing 40 μg/mL PI (Sigma-Aldrich) and 100 μg/mL RNAseA (Amersham Pharmacia, Piscataway, NJ, USA) and incubated for 30 min at 37 °C. Cell cycle analysis was performed by flow cytometry using a FACSCalibur flow cytometer (BD Biosciences) and ModFit LT Version 2.0 software (Verity Software House, Topsham, ME; RRID: SCR_016106).

1.16. Subcellular fractionation

The subcellular fractionation was performed according to the described protocol.[^6^](#_ENREF_6) 4 x 10^6^ cells were washed with PBS and resuspended in 100 µl hypotonic buffer (20 mM Tris-HCl, pH 7.5, 2 mM EDTA) supplemented with protease inhibitor mixture tablet (cOmplete, Roche). Lysates were shock-freezed in liquid nitrogen, followed by incubation on ice for 1.5 hours and physical disruption of the cells by passing through a 27G needle. After centrifugation (300 g, 10 minutes, 4 °C), the supernatant containing the cytoplasmic extract was collected. The pellet containing the nuclear extract was washed with 50 µl hypotonic buffer and lysed in 50 µl RIPA buffer (50 mM Tris-HCl, pH 7.8, 150 mM NaCl, 1 % Triton X-100, 1 % sodium deoxycholate, 250 µM EDTA). Cell lysates were separated using a 13.5 % SDS gel and transferred to a 0.2 µm nitrocellulose membrane.

1.17. Intracellular FOXO1 staining

1 x 10^6^ cells were washed with PBS, and fixed in 10 % Formalin for 10 minutes at 37 °C. Following an additional washing step in PBS, cell permeabilization was performed by adding 90 % ice-cold methanol dropwise to the sample during vortexing and incubation on ice for 30 minutes. Sample was washed with wash buffer (PBS containing 1 % FBS and 0.1 % sodium azide) and resuspended in 100 µl wash buffer containing a 1:100 dilution of FOXO1 antibody (#2880, Cell Signaling Technology, Danvers, MA, USA; RRID: AB_2106495). After incubating the sample on ice for one hour, cells were washed in wash buffer and resuspended in 100 µl wash buffer containing a 1:100 dilution of fluorochrome-conjugated secondary antibody (donkey anti-rabbit, Alexa Fluor 488, #A-21206, Thermo Fisher; RRID: AB_2535792). Cells were washed with wash buffer, resuspended in 400 µl PBS and analyzed by flow cytometry (FACSCanto, BD Biosciences).

1.18. Cell surface staining

1 x 10^6^ cells were washed with wash buffer (PBS, 0.5 % BSA, 2 mM EDTA) and resuspended in 100 µl wash buffer containing a 1:20 dilution of CXCR4-APC antibody (#17999941, Thermo Fisher; RRID: AB_1724115) or IgG2ak-APC isotype control (#17472441, Thermo Fisher; RRID: AB_10598641). After an incubation for 10 minutes at 4 °C in the dark, cells were washed with wash buffer and analyzed by flow cytometry (FACSCanto, BD Biosciences). Dotplots and histograms were created using the Flow Jo Software (RRID: SCR_008520).

1.19. Cell viability analysis

Drug sensitivities were determined by the cell viability MTT assay. For MTT assays, cells were seeded in triplicates into 96 well plates at a density of 1 x 10^5^ cells per well in complete medium. Cells were treated with 2-fold serial dilutions of AS1842856 (Merck, Darmstadt, Germany) with the highest concentration of 20 µM. Solvent control wells were incubated with dimethyl sulfoxide (DMSO), with volume corresponding to highest drug treatment. Positive control wells were treated with 5 µg/ml puromycin (#540222, Merck). Cells were incubated at standard conditions for 5-6 days, followed by addition of 25 µl of the 5 mg/ml MTT solution (Thiazolyl Blue Tetrazolium Bromide, Sigma-Aldrich) and incubation for 2 hours at 37 °C. Then, 100 µl lysis buffer (20% SDS, 50% dimethylformamide, 2 % acetic acid, 0.15 mM HCl, pH 4.7) was added and after an overnight incubation at 37 °C, the optical densities (OD) were measured at 570 nm wavelength using the SpectraMax 250 microplate reader (Molecular Devices, San Jose, CA, USA) with help of the SoftMax Pro 3.0 software (Molecular Devices; RRID: SCR_014240). Percentage of growth inhibition at a given drug concentration was calculated as (1 - OD_drug_ - OD_puromycin_) / OD_DMSO_ * 100. The half maximal inhibitory concentration was calculated using GraphPad Prism software (RRID: SCR_002798).

1.20. Immunofluoresence staining

BL tumor samples were obtained from the Institute of Pathology, University of Ulm. Four cases of BL were included in this study. BL diagnosis was in accordance with the current World Health Organization classification.[^7^](#_ENREF_7) All samples were drawn from our archive of formalin-fixed, paraffin-embedded tissues and pseudonymized to comply with the German law for ethical usage of archival tissue for clinical research (Deutsches Ärzteblatt 2003; 100 A1632). Approval for these studies was obtained from the University of Ulm ethics board. BL tumor samples were deparaffinized, followed by heat-induced epitope retrieval in Tris/EDTA buffer with pH 9.0 using a microwave (15 minutes at 360 W). To permeabilize the tissue, samples were incubated with 0.1 % Triton X-100/PBS, washed with PBS and blocked for 1 h at room temperature (RT) with 10 % donkey serum (#166643, Abcam) in PBS. Then rabbit-FOXO1 antibody (FKHR H-128, #11350, Santa Cruz; RRID: AB_640607) diluted 1:50 in 10 % donkey serum/PBS was added and incubated for 1 h at RT, followed by incubation with secondary antibody (donkey anti-rabbit, Alexa Fluor 488, #A-21206, Thermo Fisher; RRID: AB_2535792) diluted 1:200 in 10 % donkey serum/PBS for 50 min at RT.

For nuclear labeling, propidium iodide (PI) diluted in PBS was added at a concentration of 1 µg/ml and incubated for 10 seconds. The samples were embedded in Mowiol (Sigma-Aldrich). Images were acquired with the Keyence BZ-9000 (BIOREVO) microscope and the BZ-II Viewer software, and processed with ImageJ software (https://imagej.nih.gov/ij/, RRID: SCR_003070).

For immunofluorescence staining of BL cell lines, 1 x 10^5^ cells were transferred to poly-L-lysine coated glass slides by spinoculation at 600 rpm for 5 minutes. Subsequently cells were fixed for 15 minutes in 3.7 % formaldehyde, followed by permeabilization with 0.02 % NP-40/PBS for 1 minute and washing with PBS. Blocking was done by incubating the slides with 50 % FBS in PBS for 1.5 h at 37 °C. Then rabbit-FOXO1 antibody (FKHR H-128, #11350, Santa Cruz; RRID: AB_640607) diluted 1:50 in 1 % BSA + 0.2 % Triton X-100/PBS was added and incubated for 2 h at 37 °C, followed by incubation with secondary antibody (donkey anti-rabbit, Alexa Fluor 488, #A-21206, Thermo Fisher; RRID: AB_640607) diluted 1:200 in 1 % BSA + 0.2 % Triton X-100/PBS for 40 min at RT. Nuclear counterstaining as well as visualization was done as described above.

1.21. Luciferase Assay

Namalwa cells stably expressing a NF-κB-dependent luciferase reporter (3× κB.luc) containing three copies of the κB motif immediately upstream of the β-globin TATA box[^8^](#_ENREF_8) were treated for 3 days with 50 nM AS1842856 or transduced with lentiviral vectors expressing F1sh or scrambled control and FACS sorted 4 days post transduction.

1 x 10^6^ cells were washed with PBS and lysed in 100 µL lysis buffer (100 mM KPO_4_ (pH 7.4), 0.2 % Triton X-100). After incubation on ice for 5 minutes, 50 µl Luciferase-Assay buffer (20 nM Tricine, 1.07 mM MgCO_3_MgOH_2_x5H_2_0, 2.7 mM MgSO_4_, 0.1 mM EDTA, 30 mM DTT, 0.27 mM Coenzym A, 0.5 mM Luciferin, 0.53 mM ATP) was added followed by luminescence measurement on the Lumat LB 9507 tube luminometer (Berthold Technologies, Germany). Protein concentration at 280 nm was measured by Nanodrop 1000 spectrophotometer (Thermo Fisher Scientific, USA) and luciferase values were normalized to protein amount.

**Table 1.** N-terminal *FOXO1* hotspot mutations in BL cell lines.

| **Cell line ID** | ***FOXO1* Missense Mutation** |
| --- | --- |
| Namalwa | T24I |
| Jiyoye | S22P |
| Ramos | wt |
| BL-41 | wt |
| Daudi | wt |
| Raji | wt |

Summary of *FOXO1* gene sequencing results in human BL cell lines. N-terminal part of Exon 1 was analyzed, since the majority of the recurrent hot-spot mutations found in BL and DLBCL affect the consensus [RxRxxT] PKB/AKT recognition motif surrounding the T24 phosphorylation site.[^9^](#_ENREF_9)^,^[^10^](#_ENREF_10)

**Supplemental Figures and Legends**


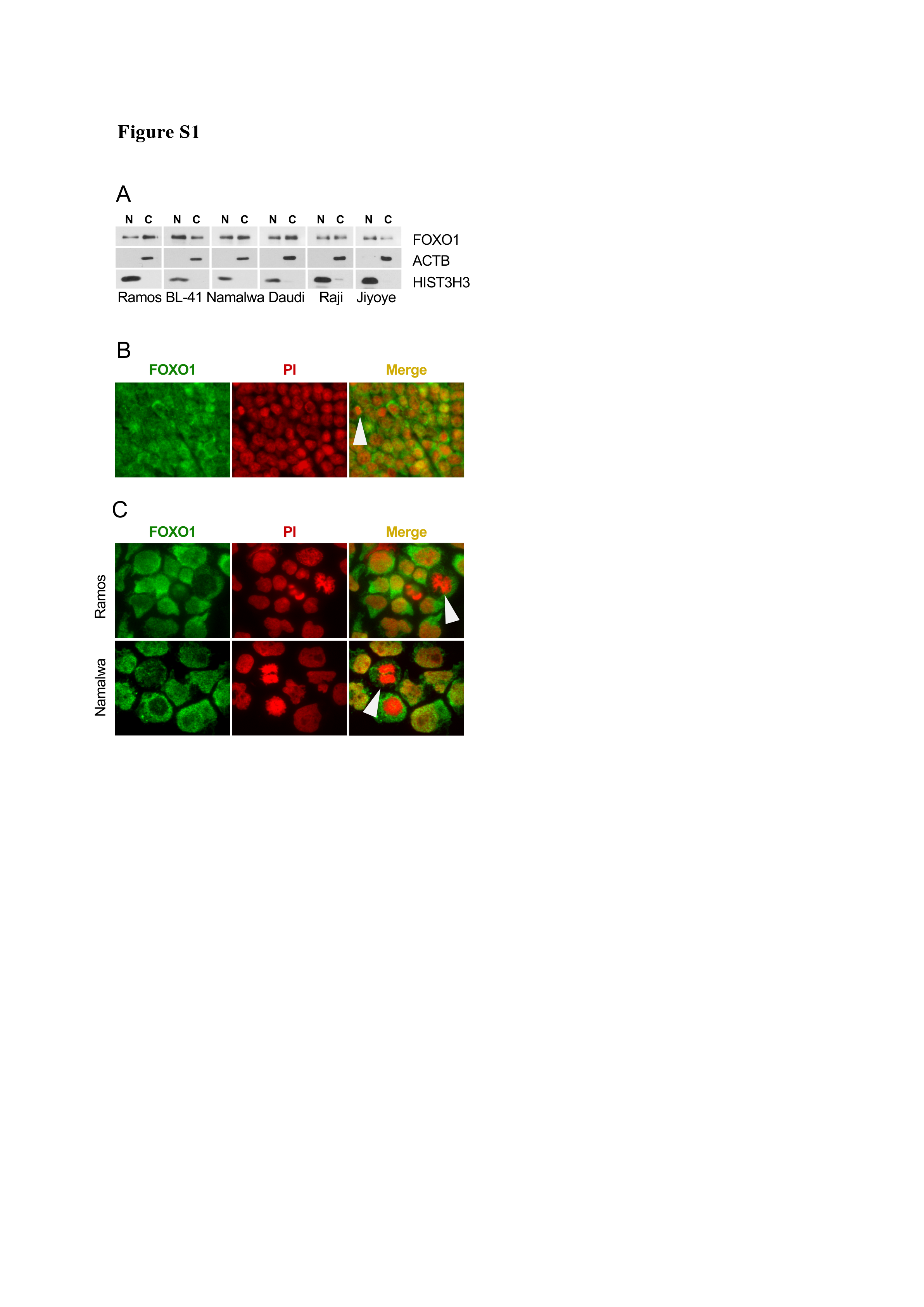


**Figure 1.** Nuclear localization of FOXO1 in BL cell lines and tumors. **(A–C) Subcellular distribution of FOXO1 in BL cell lines and samples.** (**A**) Cellular fractionation of BL cell lines was followed by immunoblotting for FOXO1. Purity of the cytoplasmic and nuclear fraction was controlled by cytoplasmic ACTB and nuclear HIST3H3 expression. A representative of two or three independent experiments is shown. (**B, C**) Immunofluoresence staining of FOXO1 in BL patient samples and BL cell lines. BL tissue sections were deparaffinized (**B**), BL cells were attached to glass slide by low speed centrifugation (**C**) and stained with FOXO1 antibody (green). Nuclei were counterstained with PI (red). Arrow indicates mitotic cell in which FOXO1 is not bound to the condensed mitotic chromatin. The images were acquired with help of the Keyence BZ-9000 (BIOREVO) microscope and BZ-II Viewer software and processed with ImageJ software. Real magnification is 308 x. (**B**) A representative of four biological replicates is shown. (**C**) A representative of two independent experiments is shown.


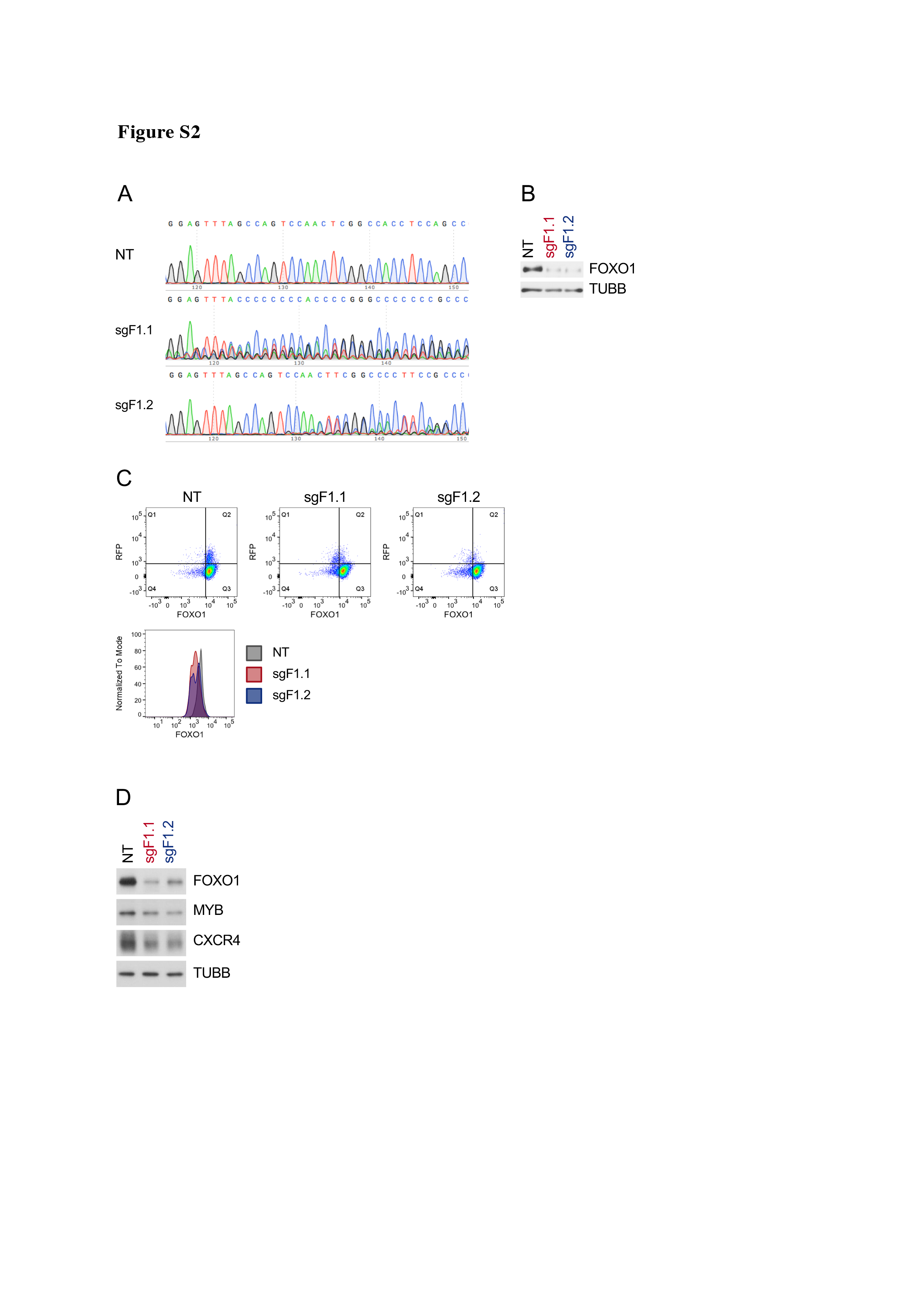


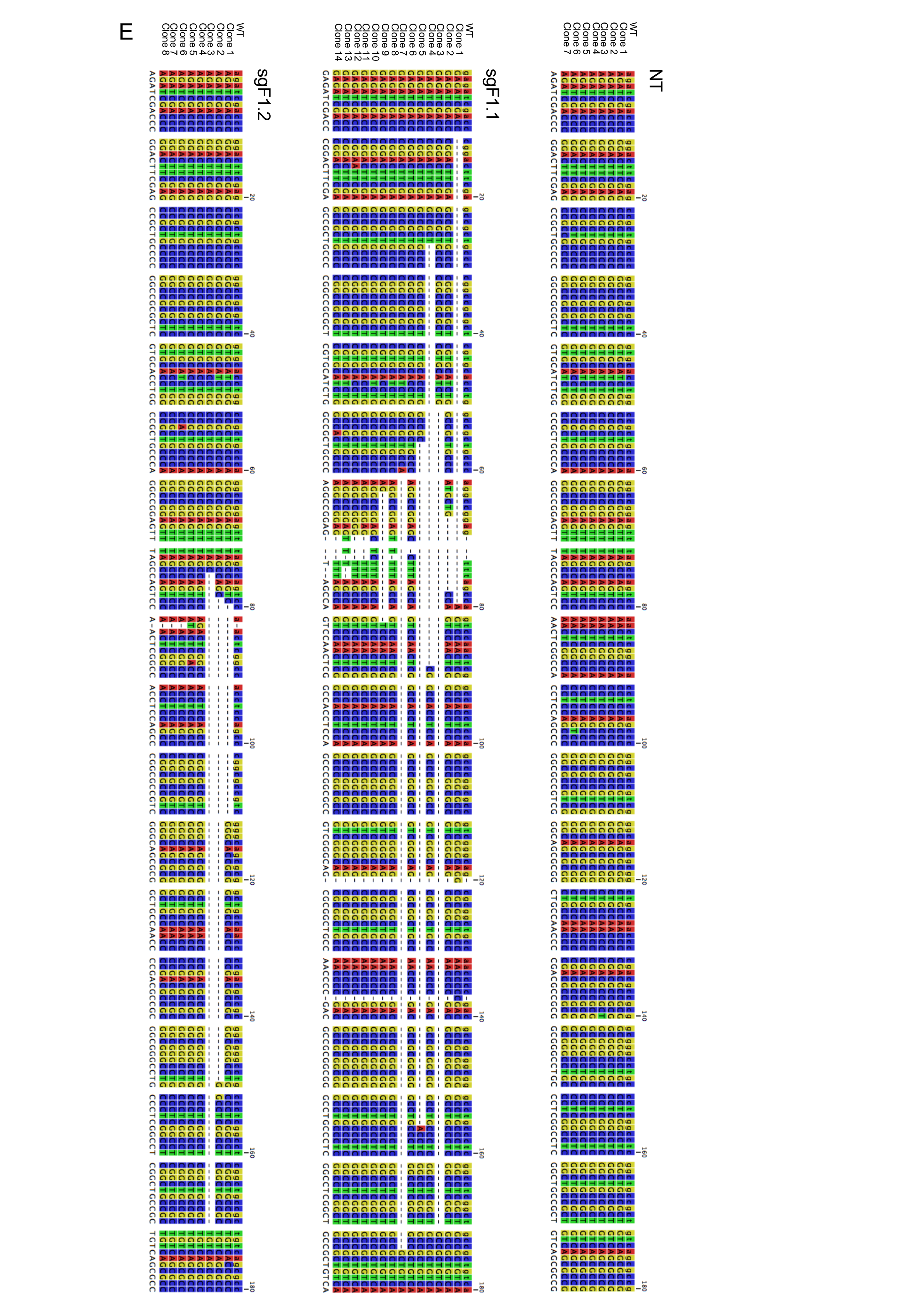


**Figure 2.** Validation of CRISPR/Cas9-mediated FOXO1 knockout in BL cell lines. (**A–D**) BL cell lines were transduced with lentiviral constructs co-expressing Cas9 with sgRNAs targeting *FOXO1* (sgF1.1, sgF1.2) or non-targeting control (NT). (**A**) Transduced Namalwa cells were FACS sorted, followed by mutational analysis of sgRNA target regions within the *FOXO1* gene compared to the NT control. (**B**) Transduced Namalwa cells were FACS sorted, followed by immunoblot confirming downregulation of FOXO1 protein levels by CRISPR/Cas constructs. TUBB served as loading control. Representative image of two independent experiments is shown. (**C**) Intracellular FOXO staining. Specificity of FOXO1 knockout was validated by intracellular staining for FOXO1 in BL-41 cell line 4 days after transduction. For histogram only transduced RFP^+^ cells were included. (**D**) Transduced Namalwa cells were FACS sorted 5 days post transduction, followed by immunoblot confirming downregulation of FOXO1, MYB and CXCR4 protein levels by CRISPR/Cas constructs. TUBB served as loading control. Representative image of two independent experiments is shown. (**E**) Characterization of the mutant alleles by sequencing of cloned PCR-amplified sgRNA target regions. The FOXO1 reference sequence is shown on top (WT). Detailed information is described in Supplemental methods.

**
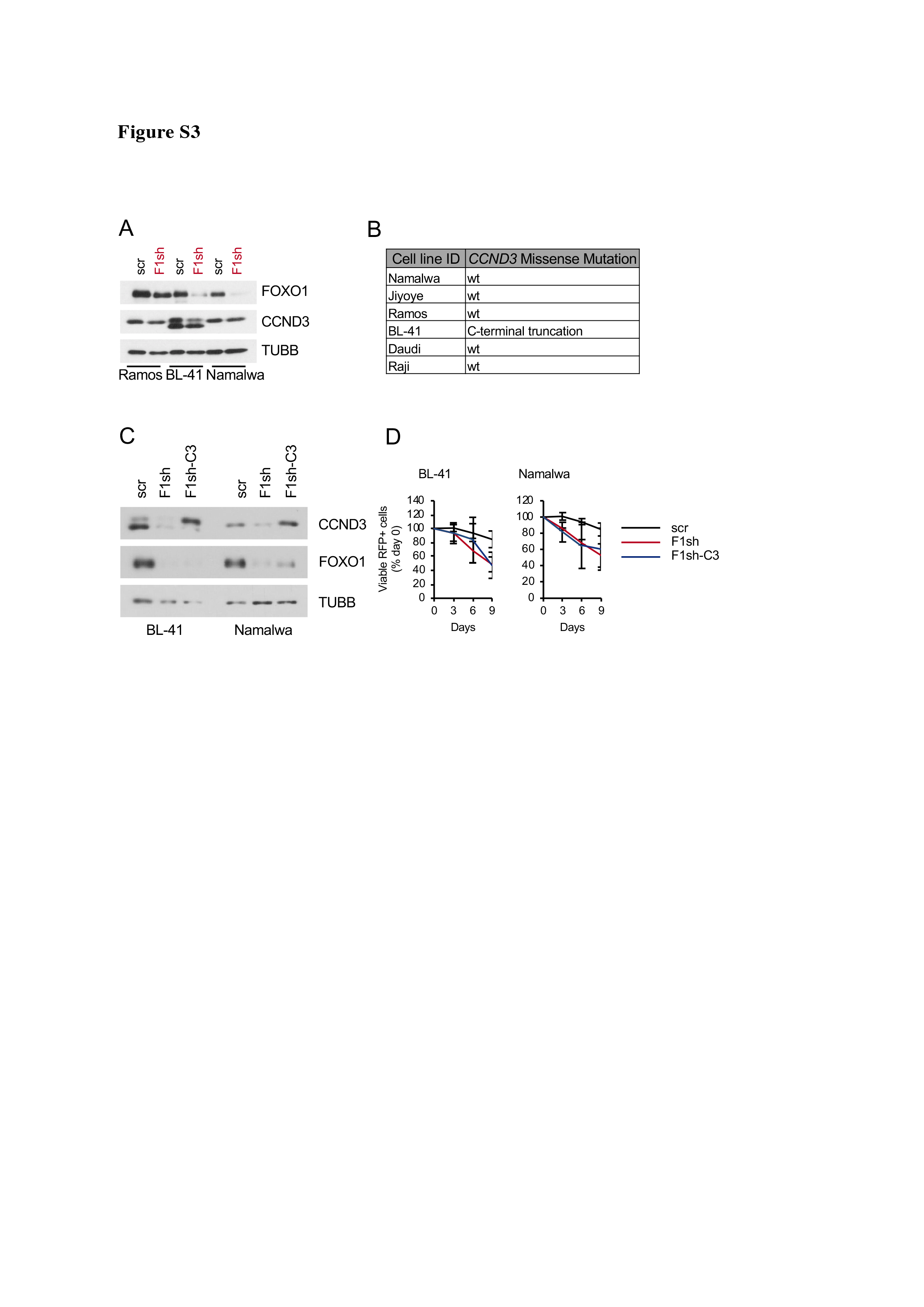
**

**Figure 3. CCND3 overexpression cannot rescue BL cells from the toxic effect of FOXO1 knockdown.** (**A**) Immunoblot of CCND3 levels after FOXO1 knockdown (F1sh) vs. scrambled control (scr). Cells were sorted and lysed 4 days post transduction. TUBB served as loading control. Representative image of three independent experiments is shown. (**B**) Mutational analysis of the CCND3 gene by cDNA-based sequencing of the CDS. (**C, D**) Expression of CCND3 does not rescue BL cells from inhibition of proliferation induced by FOXO1 knockdown. BL cell lines were transduced with a vector expressing F1sh, CCND3 and F1sh (F1sh-C3), or scr control. (**C**) Cells were sorted and lysed 5 days post transduction and the expression of FOXO1 and CCND3 was analyzed by immunoblot. TUBB served as loading control. A representative of two independent experiments is shown. (**D**) The percentage of RFP^+^ cells was measured every 3 days using flow cytometry. First measurement was performed 4 - 5 days post transduction and the percentage of RFP^+^ cells was set as 100. Data are shown as mean ± SD (N≥2).


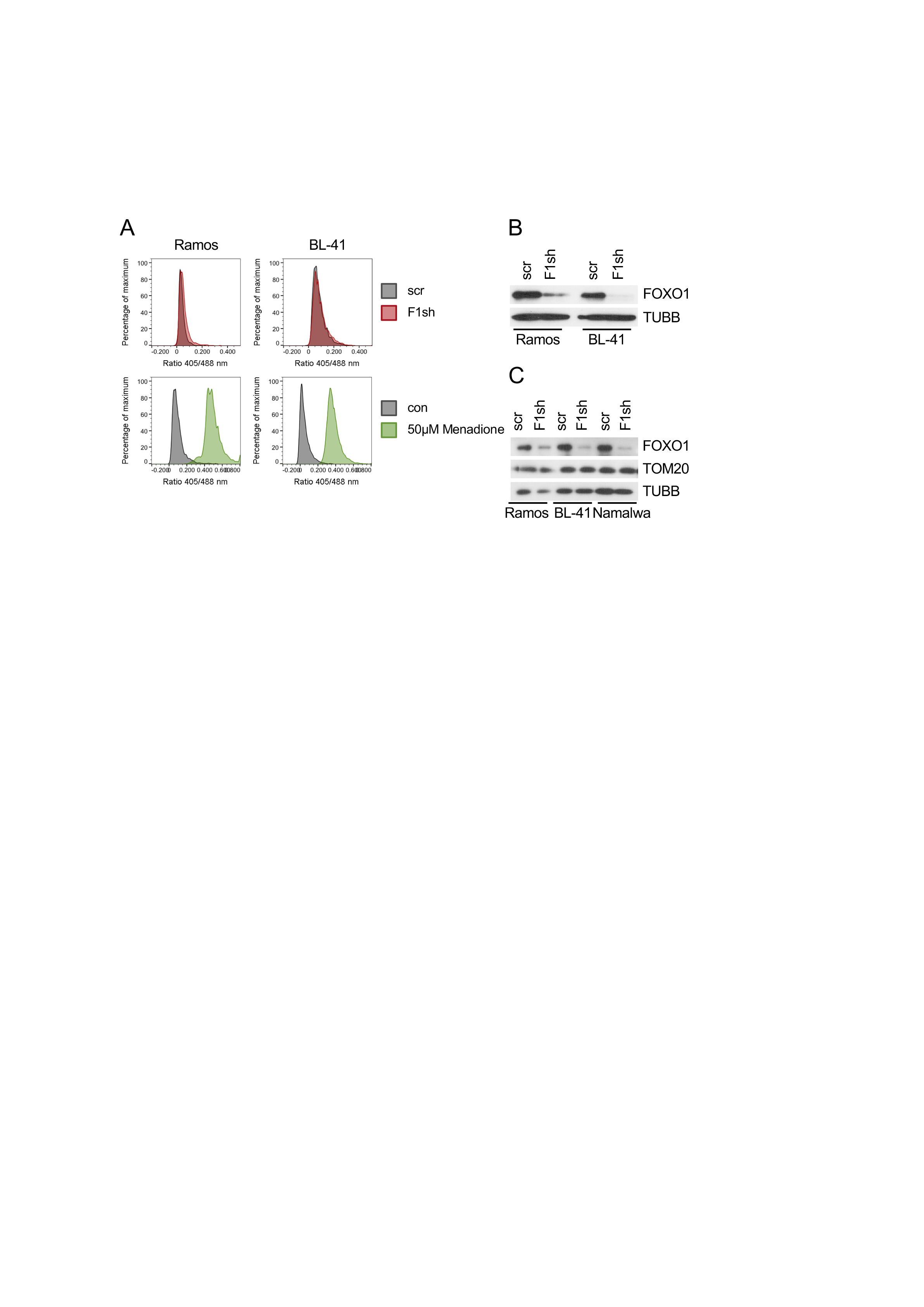


**Figure 4.** FOXO1 knockdown does not induce oxidative stress or mitophagy. (**A, B**) BL cell lines were transduced with lentiviral constructs co-epressing Grx1-roGFP2 with FOXO1 shRNA (F1sh) or scrambled (scr) control. (**A**) Redox response of BL cells to FOXO1 knockdown compared to scr control. Cells were analyzed by measuring the 405/488nm emission ratio by flow cytometry. As positive control cells were treated for 2 hours with 50 µM Menadione at 37 °C, prior to analysis. Representative image of two independent experiments is shown. (**B**) Immunoblot confirming FOXO1 downregulation in cells expressing F1sh vs. scr control. TUBB served as loading control. Representative image of two independent experiments is shown. (**C**) Immunoblot of TOM20 levels after FOXO1 knockdown vs. scr control. TUBB served as loading control. Representative image of two independent experiments is shown.

**
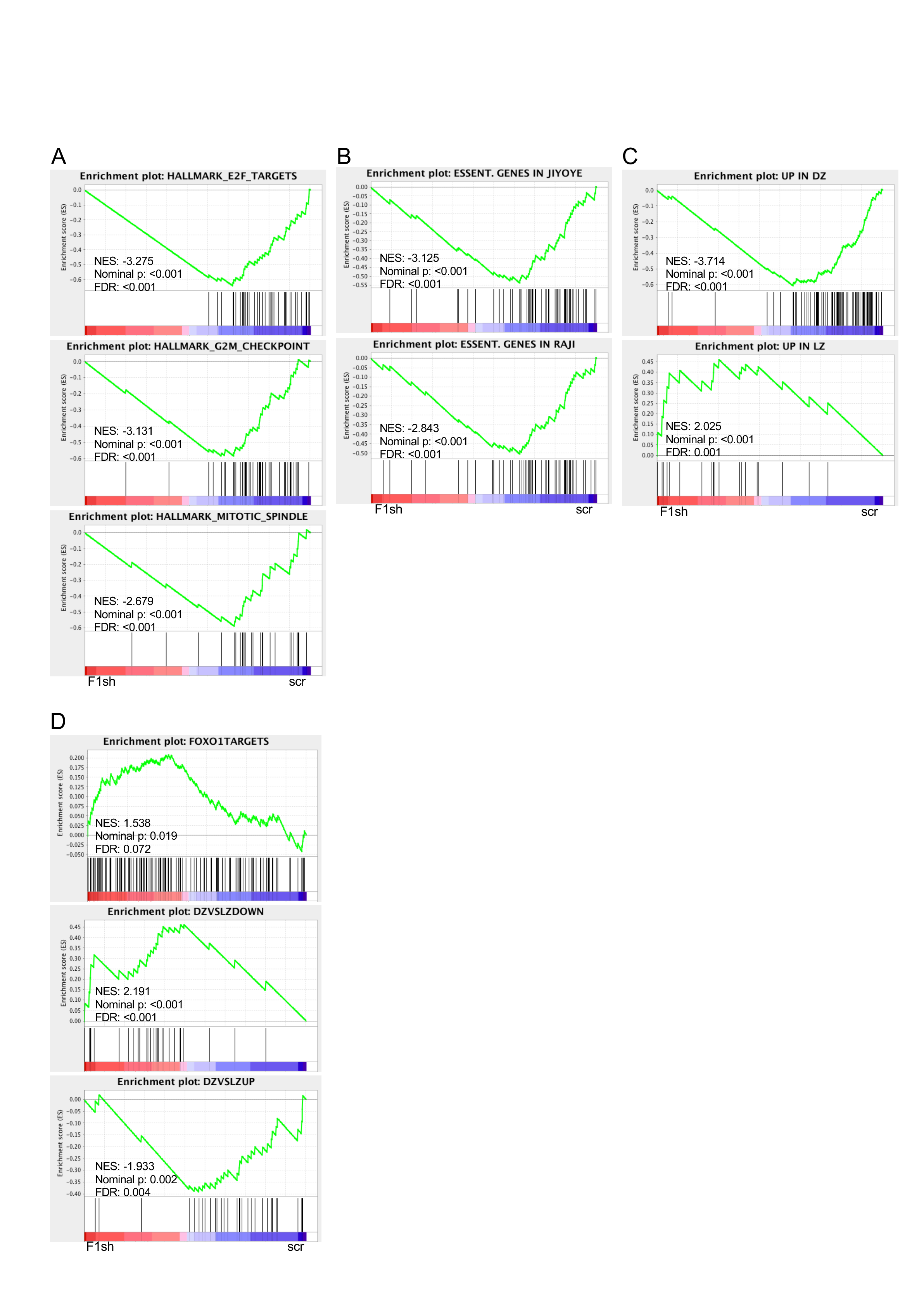
Figure 5.** FOXO1 knockdown represses the dark zone signature, proliferation associated signatures and FOXO1 target genes of germinal center dark zone B cells. (**A**–**D**) GSEA (http://software.broadinstitute.org/gsea/msigdb/index.jsp; 01-15-2017). Direction of phenotype comparison: “F1sh vs. scr”. NES: Normalized enrichment score. FDR: False discovery rate. (A) Depletion of proliferation-associated MSigDB signatures by FOXO1 knockdown: HALLMARK E2F TARGETS, HALLMARK G2M CHECKPOINT and HALLMARK MITOTIC SPINDLE (Tables S4 – S6). (B) Genes essential for survival of Jiyoye and Raji cell lines^10^ are repressed by FOXO1 knockdown (Tables S10 and S11). Gene signatures “ESSENT. GENES IN JIYOYE” or “ESSENT. GENES IN RAJI” comprise genes with CRISPR score CS >-1 (Table S19). (C) DZ and LZ signatures are repressed or induced by FOXO1 knockdown, respectively. Genes upregulated in DZ or LZ populations^11^ sorted from human tonsils were applied on set of genes modulated more than 1.5 fold by FOXO1 knockdown (threshold of 1.5, ANOVA, Benjamini and Hochberg correction, adjusted P<0.05) (Tables S15 and S16). (**D**) Top: FOXO1 target genes in human germinal center B cells were applied to a set of genes modulated more than 1.5 fold by FOXO1 knockdown^12^ (threshold of 1.5, ANOVA, Benjamini and Hochberg correction, adjusted P<0.05) (Tables S7 – S9). Middle and bottom: FOXO1 target genes differentially expressed in the GC DZ vs. LZ^12^ or vice versa were applied to the same set of genes (Table S20).

**
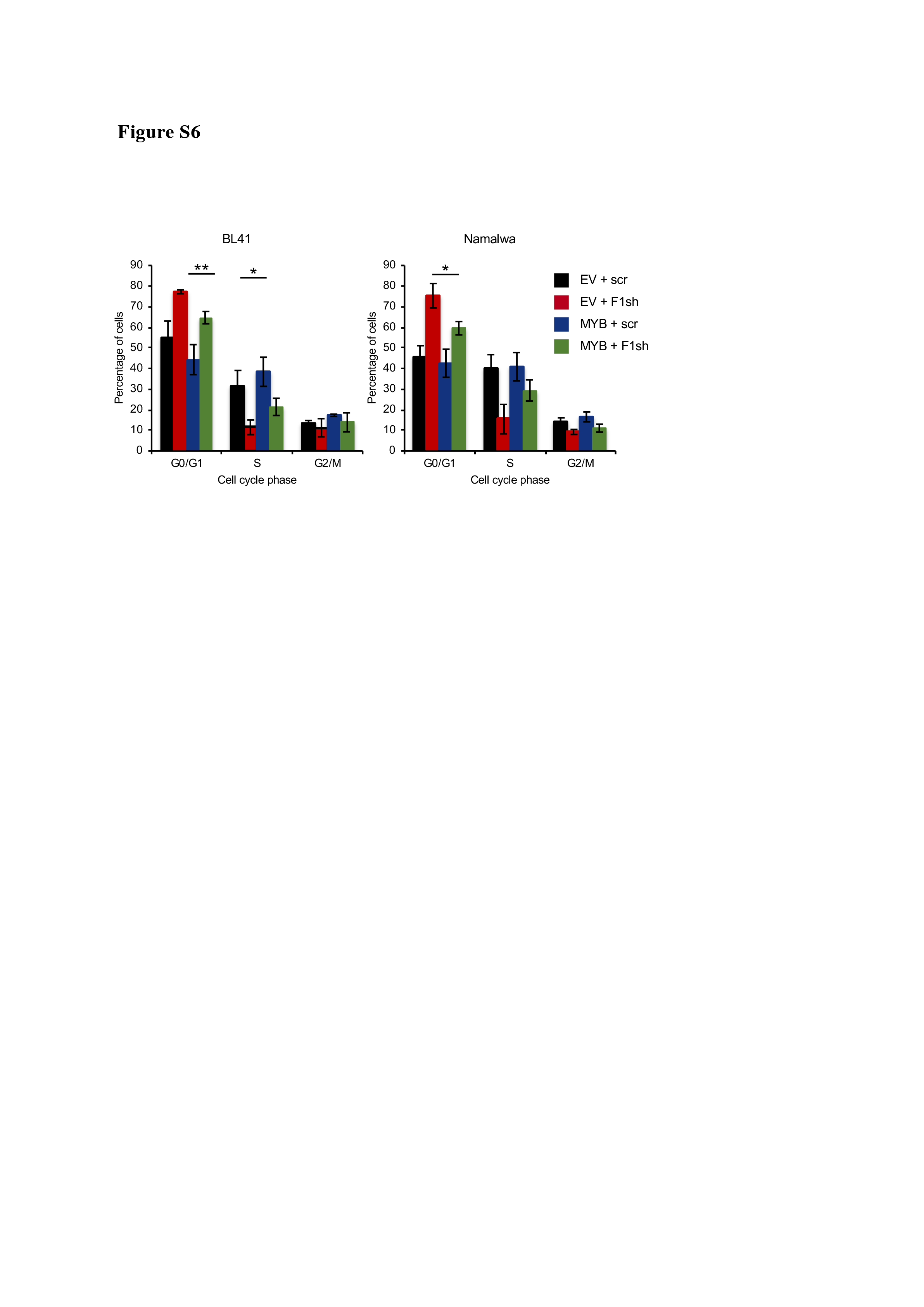
**

**Figure 6.** MYB expression partially rescues the FOXO1-knockdown induced G1 cell cycle arrest. BL cell lines expressing MYB or empty vector (EV) were transduced with F1sh vs. scr control. GFP^+^/RFP^+^ cells were sorted 4 days post transduction, followed by cell cycle analysis by PI staining. Data are shown as mean percentage of cells in a cell cycle phase ± SD (N=3). Significances between EV + F1sh and MYB + F1sh were analyzed by two-sided T-test. *, P < 0.05, **, P < 0.01.

References

1. Mader A, Bruderlein S, Wegener S, et al. U-HO1, a new cell line derived from a primary refractory classical Hodgkin lymphoma. *Cytogenet Genome Res*. 2007;119(3-4):204-210.
2. Sarbassov DD, Guertin DA, Ali SM, Sabatini DM. Phosphorylation and regulation of Akt/PKB by the rictor-mTOR complex. *Science*. 2005;307(5712):1098-1101.
3. Wang J, Sun Q, Morita Y, et al. A differentiation checkpoint limits hematopoietic stem cell self-renewal in response to DNA damage. *Cell*. 2012;148(5):1001-1014.
4. Wang F, Demir S, Gehringer F, et al. Tight regulation of FOXO1 is essential for maintenance of B-cell precursor acute lymphoblastic leukemia. *Blood*. 2018.
5. Vogel MJ, Xie L, Guan H, et al. FOXO1 repression contributes to block of plasma cell differentiation in classical Hodgkin lymphoma. *Blood*. 2014;124(20):3118-3129.
6. Chan JS, Lee JW, Ho MK, Wong YH. Preactivation permits subsequent stimulation of phospholipase C by G(i)-coupled receptors. *Mol Pharmacol*. 2000;57(4):700-708.
7. Swerdlow SH, Campo E, Pileri SA, et al. The 2016 revision of the World Health Organization classification of lymphoid neoplasms. *Blood*. 2016;127(20):2375-2390.
8. Maier HJ, Marienfeld R, Wirth T, Baumann B. Critical role of RelB serine 368 for dimerization and p100 stabilization. *J Biol Chem*. 2003;278(40):39242-39250.
9. Schmitz R, Young RM, Ceribelli M, et al. Burkitt lymphoma pathogenesis and therapeutic targets from structural and functional genomics. *Nature*. 2012;490(7418):116-120.
10. Trinh DL, Scott DW, Morin RD, et al. Analysis of FOXO1 mutations in diffuse large B-cell lymphoma. *Blood*. 2013;121(18):3666-3674.
